# RNA-based, transient modulation of gene expression in human haematopoietic stem and progenitor cells

**DOI:** 10.1038/srep17184

**Published:** 2015-11-24

**Authors:** Yvonne Diener, Marion Jurk, Britta Kandil, Yeong-Hoon Choi, Stefan Wild, Ute Bissels, Andreas Bosio

**Affiliations:** 1 Miltenyi Biotec GmbH, Bergisch Gladbach, Germany; 2 Heart Center of the University of Cologne, Department of Cardiothoracic Surgery, Center of Molecular Medicine Cologne, University of Cologne, Cologne, Germany

## Abstract

Modulation of gene expression is a useful tool to study the biology of haematopoietic stem and progenitor cells (HSPCs) and might also be instrumental to expand these cells for therapeutic approaches. Most of the studies so far have employed stable gene modification by viral vectors that are burdensome when translating protocols into clinical settings. Our study aimed at exploring new ways to transiently modify HSPC gene expression using non-integrating, RNA-based molecules. First, we tested different methods to deliver these molecules into HSPCs. The delivery of siRNAs with chemical transfection methods such as lipofection or cationic polymers did not lead to target knockdown, although we observed more than 90% fluorescent cells using a fluorochrome-coupled siRNA. Confocal microscopic analysis revealed that despite extensive washing, siRNA stuck to or in the cell surface, thereby mimicking a transfection event. In contrast, electroporation resulted in efficient, siRNA-mediated protein knockdown. For transient overexpression of proteins, we used optimised mRNA molecules with modified 5′- and 3′-UTRs. Electroporation of mRNA encoding GFP resulted in fast, efficient and persistent protein expression for at least seven days. Our data provide a broad-ranging comparison of transfection methods for hard-to-transfect cells and offer new opportunities for DNA-free, non-integrating gene modulation in HSPCs.

Haematopoietic stem and progenitor cells (HSPCs) are the starting point for blood cell production but also the origin of many haematological disorders. Moreover, HSPCs are an attractive source to reconstitute the immune system in autoreactive diseases and to potentially support regeneration of a number of different organs^1^,^2^. In this context, molecular approaches have been studied to expand HSPCs and improve their availability for clinical application^3^,^4^. For example, antisense-mediated knockdown of the cell-cycle regulator p21^5^ as well as modulation of microRNA expression^4^,^6^,^7^ were reported to enhance the expansion of HSPCs *in vitro*. A drawback of most of these promising approaches is the use of viral vectors, as they harbour the risk of genotoxic effects^8^. This issue can be addressed by using non-integrating, RNA-based approaches which result in a transient modification of gene expression. Here, the challenge is the delivery of reagents into HSPCs as these cells have been shown to be hard to transfect with standard methods^8^.

Delivery of nucleic acids with chemical transfection reagents such as cationic lipids or polymers is based on their ability to form complexes with negatively charged nucleic acids^9^. The resulting cationic complexes can attach to the negatively charged cell surface *via* electrostatic interactions and thereby facilitate the cell entry through endocytosis. The subsequent release of the cargo from the endosomal compartment into the cytoplasm has not been completely understood so far but presumably occurs *via* destabilisation of the endosomal membrane by the cationic lipids. For cationic polymers, the so-called proton-sponge effect is thought to mediate the endosomal escape: During acidification of the endosome, the polymers bind large amounts of incoming protons. This leads to a compensatory influx of chloride ions which induce water influx *via* osmosis, resulting in the disruption of the endosome^10^. In contrast, physical transfection methods such as electroporation bypass the endosomal pathway by creating transient pores in the cell membrane and directly delivering nucleic acids into the cytosol^11^. Nucleofection is a modified electroporation strategy to deliver nucleic acids also across the nuclear membrane with high efficiency in HSPCs^12^.

We validated different methods to transiently overexpress or knockdown genes in CD133^+^ HSPCs by introducing RNA-based molecules. We compared standard liposomal transfection reagents with electroporation as well as alternative approaches such as PLGA (Poly(lactic-co-glycolic acid)) nanoparticles as delivery method. For protein knockdown, we used standard small interfering RNAs (siRNAs) whereas for overexpression of target proteins we tested optimised messenger RNAs (mRNAs) with modified 5′- and 3′-UTRs .

We could show that HSPCs were inaccessible to liposomal reagents as well as cationic polymers. Although some reagents suggested a highly efficient transfection with a non-targeting, fluorescently labelled siRNA, we observed no target knockdown using functional siRNAs. In contrast, electroporation allowed an efficient delivery of RNAs into HSPCs. Both, protein knockdown using siRNAs as well as high and persistent protein overexpression with mRNA molecules could be achieved.

## Results

### Chemical transfection of HSPCs with fluorescent siRNA

**Figure 1:**
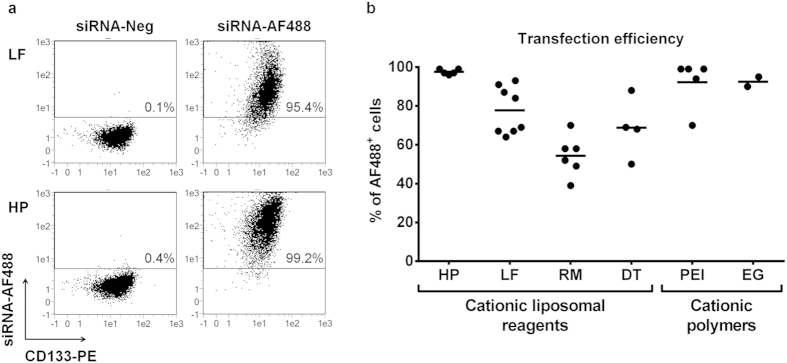
Transfection of fluorescent siRNA into CD133^+^ HSPCs with liposomal reagents and cationic polymers. Cells were analysed 19 to 24 h after transfection with different reagents and either a non-labelled, non-targeting negative control (siRNA-Neg) or a fluorescently labelled, non-targeting siRNA (siRNA-AF488). Cells were washed, stained with CD133-PE, and analysed *via* flow cytometry. (**a**) Representative dot plots after transfection with Lipofectamine 2000 (LF) and HiPerFect (HP), gated on viable cells. (**b**) Percentage of total AF488^+^ cells after optimisation of transfection conditions. Each dot represents one independent experiment (n = 2–8). RM: Lipofectamine RNAiMAX, DT: DOTAP, PEI: Polyethylenimine 2K, EG: ExGen 500.

HSPCs were transfected with ten different reagents covering three classes of chemical transfection methods. We chose seven cationic liposomal reagents, among them well established and commonly used products such as Lipofectamine 2000, RNAiMAX and DOTAP (N-[1-(2,3-Dioleoyloxy)propyl]-N,N,N-trimethylammonium methyl-sulfate). Furthermore, we tested two polyethylenimine (PEI)-based cationic polymers, which differ in their molecular weight and branching of the molecule chains. Both have been described for transfection of HSPCs, but PEI 2 K (the low molecular, branched form) showed a higher efficiency than ExGen 500, which is based on a 22 K, linear PEI molecule^13^. As a third class of chemical reagents, a calcium-phosphate-based method was tested. Transfection efficiency was determined by using a fluorescently labelled, non-targeting siRNA (siRNA-AF488, Fig. 1a). We first performed optimisation experiments titrating the amount of siRNA and reagents (Supplementary Table S1). We obtained the highest percentage of fluorescent cells (up to 99%) with the cationic lipids HiPerFect and Lipofectamine 2000 as well as with the two cationic polymers PEI 2K and ExGen 500 (Fig. 1b). The cationic liposomal reagents DharmaFECT 4, TransIT-siQUEST, Trans-IT TKO and the calcium-phosphate reagent ProFection resulted in ≤7% of AF488^+^ cells (Supplementary Table S1). Viability after treatment with transfection reagent was >97% for all tested reagents except for Lipofectamine 2000 (89%, SD ± 9), PEI 2K (83%, SD ± 14) and DOTAP (70%, SD ± 22, data not shown).

### Electroporation but not chemical transfection of siRNAs results in target knockdown

**Figure 2:**
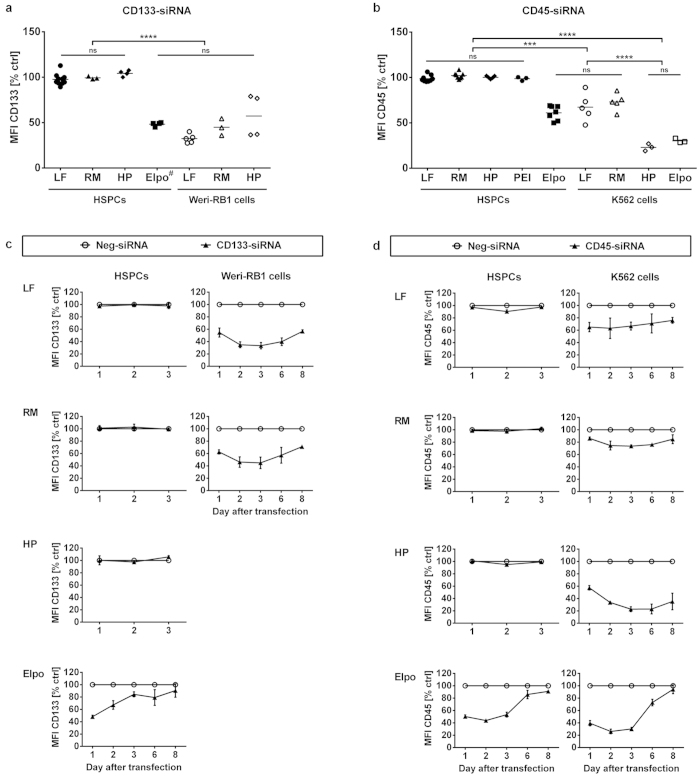
Chemical transfection of HSPCs with functional siRNAs shows no target protein knockdown but electroporation does. CD133^+^ HSPCs or indicated cell lines (Weri-RB1, K562) were transfected with siRNAs targeting CD133 (**a,c**), CD45 (**b,d**) or a non-targeting control siRNA (ctrl). Displayed is the median fluorescence intensity (MFI) of the respective surface marker as percentage relative to the control siRNA (**a,b**) Relative protein expression at the time of maximum knockdown (72 h after transfection, (^#^) 24 h after transfection; n = 2–10). (**c,d**) Kinetics of relative protein expression at the indicated time points after transfection. Open circles (О) represent cells transfected with non-targeting control siRNA (Neg-siRNA), closed triangles (▲) represent CD133- and CD45-siRNA in (**c,d**), respectively (mean of n = 3 at day 1–3, mean of n = 2 at day 6 and 8). LF: Lipofectamine 2000, RM: Lipofectamine RNAiMAX, HP: HiPerfect, Elpo: Electroporation, PEI: Polyethylenimine 2 K. Error bars represent SD. ***P ≤ 0.001, ****P ≤ 0.0001, ns = not significant, one-way ANOVA.

To validate the siRNA delivery, we transfected cells with non-labelled, functional siRNAs against cell surface proteins, allowing an easy flow cytometric readout of the potential knockdown. As target proteins, we chose either CD133 (PROM1), a stem cell marker which is present on primitive HSPCs and down-regulated during differentiation^14^ or CD45 (PTPRC), an ubiquitous nucleated blood cell marker which shows moderate expression on HSCs (haematopoietic stem cells)^15^. As control, we used a non-targeting scrambled siRNA (Neg-siRNA) of which the sequence is not complementary to any known mRNA, so it is expected to have no effect on the cells. Transfection of CD133^+^ HSPCs with CD133-siRNA or CD45-siRNA did not lead to a knockdown of the respective target protein compared to a non-targeting control siRNA, with any of the tested reagents (Fig. 2a,b). The protein expression was analysed at different time points and no knockdown was detectable between 24 hours and three days (Fig. 2c,d, left panels). The corresponding absolute median fluorescence intensities are displayed in Supplementary Figure S1. qRT-PCR analysis after 48 hours confirmed that there was no effect of the siRNAs at the mRNA level (Supplementary Fig. S2). When using the same siRNAs and reagents for transfection of the CD133-expressing cancer cell line Weri-RB1 or the CD45-expressing haematopoietic cell line K562, there was a significant knockdown of the respective target protein (P ≤ 0.001), proving the functionality of siRNAs and reagents (Fig. 2a,b). Analysis of knockdown kinetics revealed the strongest knockdown two to three days after transfection (Fig. 2c,d, right panels). However, the maximum knockdown efficiency in control cell lines was only 68% (SD ± 5.0, n = 5) and 77% (SD ± 3.9, n = 3) for Weri-RB1 and K562 cells, respectively. In order to investigate the responsiveness of the surface marker expression to translational repression, we performed protein stability assays using the protein synthesis inhibitor Cycloheximide (CHX). Upon treatment with 100 μg/mL of CHX for 31 hours, the expression of CD133 and CD45 on HSPCs decreased to 40 and 50% of untreated control cells, respectively. In K562 cells, CD45 expression decreased to around 58% of control, indicating that the surface proteins are highly stable and this limits the achievable maximum knockdown (data not shown).

Based on the results of the transfection experiments, we concluded that HSPCs must have certain properties which make them inaccessible to chemical transfection methods. We therefore tested electroporation as an alternative, physical transfection method. We electroporated HSPCs with CD133-, CD45- or non-targeting control siRNA (Neg-siRNA). The expression of the respective target protein significantly decreased to around 48% (SD ± 2.2, n = 4) with CD133-siRNA and 61% (SD ± 7.9, n = 7) with CD45-siRNA compared to control cells (Fig. 2a,b, P ≤ 0.001). The knockdown efficiency was comparable to results from the lipofection of the control cell lines Weri-RB1 and K562 (Fig. 2a,b). The maximum knockdown was detected two to three days after transfection except for electroporation of HSPCs with CD133-siRNA, which showed the greatest knockdown already after 24 hours. This is due to the endogenous downregulation of CD133 expression in the negative control during cultivation of HSPCs, as shown in Supplementary Fig. S1a. Viability of the cells 24 hours after electroporation decreased to 43% (SD ± 4.7, n = 4) with CD133-siRNA and 40% (SD ± 5.4, n = 7) with CD45-siRNA. Electroporation of fluorescently labelled, non-targeting siRNA resulted in 92.4% (SD ± 5.3, n = 4) AF488^+^ cells (data not shown).

As an alternative approach to deliver RNA-based molecules into HSPCs, we tested PLGA nanoparticles. We encapsulated CD45-siRNA *via* double emulsion technique as described in the Supplementary Methods section^16^,^17^. As a positive control, we added PLGA nanoparticles to CD14^+^CD45^+^ monocytes, which have been shown to excessively internalise the particles *via* phagocytosis^18^. After 72 hours, CD45 protein expression of monocytes decreased to around 78% of control cells which were cultivated with empty nanoparticles, showing that the siRNA was successfully encapsulated and the particles were taken up by the monocytes (Supplementary Fig. S3a). Cultivation of HSPCs with the CD45-siRNA particles showed no effect on target protein expression (Supplementary Fig. S3a,b), while electroporation of the CD45-siRNA particles resulted in a considerable CD45 knockdown. We concluded that, just as with liposomal reagents, either HSPCs do not efficiently take up the particles, or the particle cargo is not released from the endosome. Furthermore, electroporation of encapsulated siRNA did not further enhance knockdown efficiency or duration compared to electroporation of siRNA alone (Supplementary Fig. S3b). In conclusion, electroporation of siRNA alone is the most efficient method for transient gene silencing in HSPCs.

### Chemically transfected siRNA does not enter the cytoplasm of HSPCs

**Figure 3:**
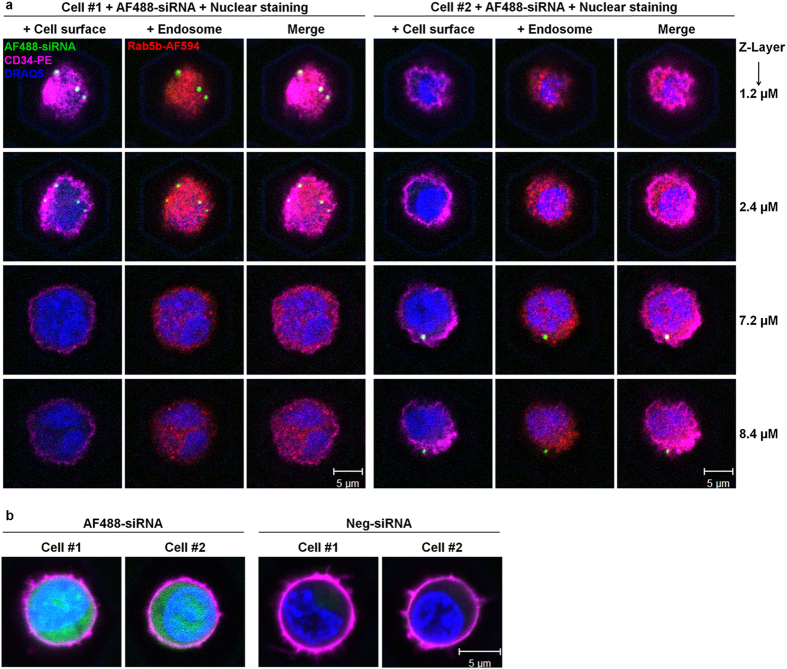
Localisation of fluorescent siRNA after transfection. (**a**) Chemical transfection: CD133^+^ HSPCs were transfected with siRNA-AF488 (green) using Lipofectamine 2000. After 24 h, cells were washed, and stained with the DNA dye DRAQ5 (blue), the cell surface marker conjugate CD34-PE (magenta) and the endosome marker conjugate Rab5b-AF594 (red). Rows show representative pictures of four different cell layers from top to bottom (Z-Stack), *left* and *right panel* displaying two individual representative cells (#1 and #2). (**b**) Electroporation of two representative cells (#1 and #2) with fluorescent AF488-siRNA (*left panel*) and non-labelled Neg-siRNA (*right panel)*, analysed and stained with DRAQ5 (blue) and CD34-PE (magenta) after one hour.

Our chemical transfection experiments indicated that the siRNAs were internalised into the cell, as the cells became AF488^+^ with the fluorescently labelled siRNA. However, they could not exert their function, as we saw no knockdown with functional siRNAs. We hypothesised that siRNAs were captured inside the endosomal compartment after chemical transfection. This is a known risk of chemical transfection methods as the siRNA-reagent complexes are internalised *via* endocytosis and thereby first localise to the endosomal compartment. From here, the siRNA has to be released into the cytoplasm, where it can assemble with the RNA-induced silencing complex to exert its function. We performed co-localisation experiments using confocal microscopy of HSPCs after transfection with siRNA-AF488 (Fig. 3). The results indicate that the siRNA-reagent complexes formed large aggregates of around 0.5 μM in diameter, which were located on or inside the cell membrane (Fig. 3a). We did not observe any co-localisation with the endosomal compartment, labelled by the endosomal marker Rab5b. We conclude that the transfection complexes were not efficiently internalised. This issue is circumvented by electroporation, which allows the single siRNA molecules to directly enter the cytoplasm. Accordingly, microscopic analysis after electroporation of siRNA-AF488 showed a homogeneous fluorescence of the cytoplasm (Fig. 3b). Taken together, our experiments show that electroporation is the method of choice for siRNA-mediated protein knockdown in primary HSPCs, whereas chemical transfection reagents are not useful for this purpose.

### Chemical transfection *versus* electroporation for mRNA-based protein overexpression

**Figure 4:**
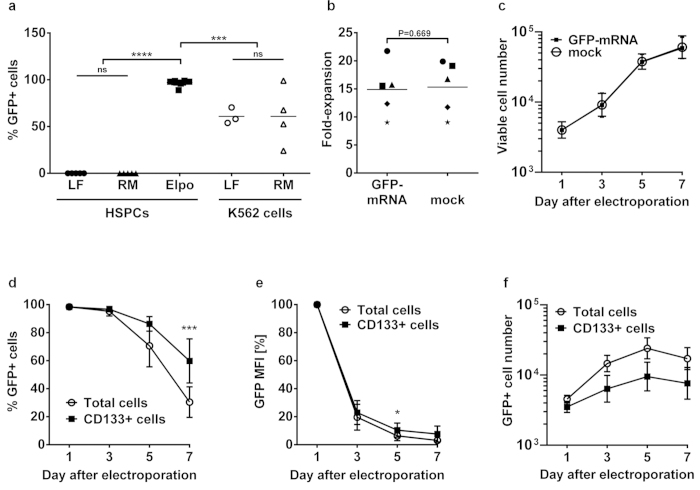
Electroporation of CD133^+^ cells with GFP-mRNA is highly efficient. (**a**) CD133^+^ HSPCs or a control cell line (K562) were transfected with a GFP-encoding mRNA using LF (Lipofectamine 2000), RM (Lipofectamine RNAiMAX) or electroporation (Elpo). GFP expression was analysed 24 h after electroporation *via* flow cytometry. Displayed is the percentage of GFP^+^ cells of three to ten independent experiments. (**b**) Fold-expansion over seven days of total cells after electroporation with GFP-mRNA or water (mock) in five independent experiments. Different symbols represent the five different donors. (**c**) Growth curves of GFP-mRNA- or mock-electroporated cells. (**d**) GFP expression among total and CD133^+^ cells at different time points after electroporation with GFP-mRNA. (**e**) Median fluorescence intensity (MFI) of GFP over time in total and CD133^+^ cells, normalised to d1. (**f**) Growth curves of total GFP^+^ and CD133^+^GFP^+^ cells after electroporation with GFP-mRNA. (**c–f**) Displayed is the mean of five independent experiments. Error bars represent SD. *P ≤ 0.05, ***P ≤ 0.001, ****P ≤ 0.0001, ns = not significant, one-way ANOVA (**a**) or paired t-test (**b,d,e**).

For DNA-free overexpression of proteins in HSPCs, we used *in vitro* transcribed optimised mRNA molecules. Here, we first tested chemical transfection reagents as well because the molecular properties of siRNA and mRNA molecules differ, e.g. in their size, and this might influence the transfection results. HSPCs were transfected with a GFP-encoding mRNA using Lipofectamine 2000 and RNAiMAX but with none of them we could detect any GFP expression (Fig. 4a, n = 5). As positive control, K562 cells were transfected with the GFP-mRNA. After 24 hours, transfection with Lipofectamine 2000 and RNAiMAX resulted in 61% (SD ± 8.6, n = 3) and 61% (SD ± 31.2, n = 4) GFP^+^ K562 cells, respectively, showing that the mRNA was functional. Electroporation of CD133^+^ HSPCs with GFP-mRNA was even more efficient, with 97% (SD ± 2.7, n = 12) GFP^+^ cells 24 hours after electroporation (Fig. 4a). Viability was 44.1% (SD ± 16.8, n = 12) after electroporation without any difference between mRNA- and mock-electroporated cells (equal volume of water instead of mRNA solution; Supplementary Fig. S4a). Furthermore, both mRNA- and mock-electroporated cells displayed the same expansion rates and growth kinetics after electroporation (Fig. 4b,c). The expression of the stem cell marker CD133 was comparable in both conditions as well (Supplementary Figure S4b). Thus, the mRNA itself is not toxic and although electroporation harms the cells, they are still functional. Remarkably, the GFP signal persisted for at least seven days after electroporation (Fig. 4d). As the frequency of CD133^+^ HSPCs decreases during cultivation, we compared the GFP expression in this subpopulation with the total cells. The CD133^+^ cells retained a significantly higher frequency of GFP^+^ cells after seven days (Fig. 4d, P = 0.004, paired t-test) as well as a significantly higher GFP protein level on day five (Fig. 4e, P = 0.01). This is probably due to the lower proliferation rate of the more primitive CD133^+^ subpopulation compared to the total cells. The absolute cell numbers of total- and CD133^+^- GFP^+^ cells increased until day five after electroporation, showing that the GFP^+^ cells are vital and able to proliferate (Fig. 4f). In summary, electroporation of optimised GFP-mRNA resulted in highly efficient, long-lasting and non-toxic protein expression in primary HSPCs.

## Discussion

Over the last twenty years, various efforts have been undertaken to successfully introduce nucleic acids into primitive HSPCs^8^. In our study, we confirmed that neither liposomal reagents nor nanoparticles were able to efficiently transfect HSPCs with RNA-based molecules. Nevertheless, there are several publications which have successfully transfected those cells under certain conditions. For example Martino and colleagues showed that the liposomal reagent DOTAP is suitable for efficient delivery of siRNA in HSPCs differentiating into dendritic cells^19^. Serial lipofection of a functional siRNA at day 0 and 3 under differentiating culture conditions led to 100% knockdown of the respective target protein at d7. This indicates that differentiating cells are much more susceptible to lipofection than quiescent HSCs. However, chemical transfection might still be acceptable for primitive cells in settings where low toxicities are of highest priority and minor efficiencies can be tolerated, for example if the resulting gene modification leads to a significant growth advantage or the successfully transfected cells can be selected afterwards. The same applies to PLGA nanoparticles as delivery vehicles. PLGA is a non-toxic, biodegradable polymer, which is FDA and EMA approved for various drug delivery applications^16^. McNeer and colleagues treated CD34^+^ cells with PLGA particles containing triplex-forming peptide nucleic acids (PNAs) and observed high levels of genomic recombination^17^. In our study, the internalisation of siRNA-loaded particles was not efficient enough to detect a protein knockdown, which presumably requires a higher intracellular concentration of the nucleic acid. The reasons why HSPCs are almost totally resistant to chemical transfection methods are still not fully understood. HSPCs have to maintain and rebuild the blood system throughout the whole life of an organism. Therefore they must have evolved mechanism which make them inaccessible to infectious and toxic influences. One important point is surely that these mostly quiescent cells have a reduced metabolic activity that allows them to decrease also their endocytic activity. Furthermore, special efflux mechanisms like multi-drug resistance transporters^20^ might contribute to the low transfection efficiencies. A further possible defence mechanism of HSCs might consist of an altered endosomal pathway, which prevents the release of the transfection cargo into the cytoplasm. Confocal microscopic analysis after chemical transfection with a fluorescently labelled siRNA suggests that the siRNA complexes mainly form large aggregates that either stick to the cell surface or inside the membrane. Presumably, these aggregates were too big to be completely internalised or the endocytic activity of the cells was too low for an efficient uptake of the complexes. However, we cannot exclude that single siRNA molecules were internalised but not released from the endosomal compartment, although microscopic co-localisation studies could not prove this hypothesis. Most probably, the number of internalised molecules was too low to emit a detectable fluorescence signal or the siRNA was already degraded at the time point of analysis. Our data demonstrate that the use of fluorescent non-targeting siRNA for assessment of transfection efficiency has to be considered with caution.

During the last decade, electroporation has been shown to be a harsh, but very efficient method to transfect also primitive, non-cycling HSCs without impairing their function^12^,^21^. The only drawback of electroporation in general is the relatively high lethality compared to lipofection, although this effect seems to be cell type specific^22^. Our results confirmed these findings and furthermore proved that electroporation is suitable for efficient delivery of small RNAs as well as mRNA molecules into CD133^+^ HSPCs, in agreement with previously published data^23^,^24^. However, in these previous studies, electroporation of GFP-mRNA either resulted in relatively low transfection efficiencies, e.g. 35% GFP^+^ cells^25^, or the GFP-mRNA decreased the viability of the cells compared to mock-electroporated cells^24^. In contrast, our modified GFP-encoding mRNA showed extremely high transfection efficiency of almost 100% after electroporation in HSPCs without any adverse effects regarding viability or proliferation.

Taken together, our study provides a broad-ranging overview about utility of different transfection methods for RNA-based molecules into HSPCs. We confirmed that chemical transfection methods are unsuitable for HSPCs and that electroporation is the method of choice for efficient transfection of HSPCs with siRNAs. In addition, we showed that carefully prepared mRNA molecules led to a highly efficient, persistent protein expression without any cytotoxicity. This offers also new opportunities for therapeutic gene modulation in HSPCs without the risk of unwanted genomic alterations.

## Methods

### Cell isolation and culture

HSPCs were isolated from fresh human cord blood or bone marrow aspirate after informed written consent using guidelines approved by the local Ethics Committee (Ärztekammer Nordrhein, Permit Number: EK103/2011) and all methods were carried out in accordance with the approved guidelines. Umbilical cord blood was obtained from healthy newborn donors after informed parental consent by puncture of the umbilical cord vein after cord clamping (Vinzenz Pallotti Hospital, Bergisch Gladbach, Germany or DKMS Nabelschnurblutbank, Dresden, Germany) and stored at room temperature with the anticoagulant heparin or CPD until usage. Bone marrow was obtained from the Department of Cardiothoracic Surgery in Cologne or purchased from All Cells. CD133^+^ HSPCs were isolated by using the CD133 Microbead Kit (Miltenyi Biotec). The purity of CD133^+^ cells was 82–97% with a median value of 94.6% (SD ± 4). Viability was above 97%. Cells were cultured at 37 °C/5% CO_2_ in StemSpan serum-free expansion medium (StemSpan SFEM, StemCell Technologies) supplemented with 10 μg/mL (1.72 IU/mL) Heparin (Ratiopharm), early acting cytokines (10 ng/mL human FGF-1, 10 ng/mL human SCF, 20 ng/mL human TPO, all from Miltenyi Biotec) and penicillin/streptomycin (PAA).

Primary monocytes were isolated from the negative fraction after CD133^+^ cell separation from cord blood using the CD14 MicroBead Kit (Miltenyi Biotec) according to the manufacturer’s instructions. Monocytes were cultivated in Mo-DC Differentiation medium (Miltenyi Biotec). K562, Molt4 and Weri-RB1 cell lines were cultivated in RPMI 1640 supplemented with 10% FCS, 2 mM L-Glutamine and penicillin/streptomycin.

### Flow cytometry

For flow cytometry, cells were stained with the following antibodies from Miltenyi Biotec: CD133/2-PE (clone: 293C3), CD34-APC (clone: AC136) and CD45-VioBlue (clone: 5B1) according to the manufacturer’s protocol (Miltenyi Biotec). To determine the cell viability, propidium iodide was added to the samples just before measurement. Flow cytometric analysis was performed using MACSQuant Analyzer 10 (Miltenyi Biotec).

### Chemical transfection of CD133^+^ cells and cell lines

Cells were transfected with HiPerFect (Qiagen), Lipofectamine 2000 (Life technologies), Lipofectamine RNAiMAX (Life technologies), DOTAP (Roche), DharmaFECT 4 (Dharmacon), TransIT-TKO (Mirus), TransIT-siQUEST (Mirus), ExGen500 (Euromedex), Polyethylenimine 2 K (Sigma-Aldrich) or ProFection (Promega) using the manufacturer’s protocols with slight modifications (see Supplementary Table 1). Transfection efficiency was assessed by using a fluorescently labelled, non-targeting siRNA (siRNA-FITC or siRNA-AF488, AllStars Negative Control siRNA with Fluorescein or Alexa Fluor 488, Qiagen) at a final concentration between 15 and 100 nM. As negative control we used non-labelled, non-targeting control siRNA, which has no known target mRNA and is supposed to have no effect on the cells (siRNA-Neg; AllStars Negative Control siRNA, Qiagen). Cells were analysed for fluorescence after 19–24 h using the MACSQuant Analyzer 10 (Miltenyi Biotec). Transfection efficiency was calculated as the percentage of fluorescent cells with regard to the total cells. The gate was set according to the negative control. Viability is given as the percentage of viable, propidium iodide-negative cells relative to the viable cell number of untreated control cells. Transfection with functional siRNAs was performed with 100 nM siRNA targeting CD133 (ON-TARGET plus siRNA PROM1, #2, target sequence: 5′-GAAGUAUGGGAGAACAAUA-3′), CD45 (ON-TARGET plus SMART pool human PTPRC, mixture of 4 siRNAs targeting the following sequences: 5′-GGCUUAAACUCUUGGCAUU-3′, 5′-AGUAUUUGUGACAGGGCAA-3′, 5′-ACUCUUGGCAUUUGGCUUU-3′, 5′-CAGAAGUAUUUGUGACAGG-3′) or ON-TARGETplus Non-targeting siRNA#1 as negative control (all from Dharmacon). Protein knockdown was assessed by flow cytometry 24 h (d1)–d8 after transfection. For protein overexpression, cells were transfected with 150 ng of GFP-mRNA per well and analysed by flow cytometry after 24 h.

For transfection experiments, overnight cultivated CD133^+^ cells were seeded into U-Bottom 96-Well plates at 5 × 10^3^ cells per well in 100–150 μL StemSpan SFEM with Heparin and cytokines. K562, Molt4 and Weri-RB1 cells were seeded at 2 × 10^4^ cells per well in the respective culture medium. RNA and transfection reagent were diluted in StemSpan SFEM or Opti-MEM (Life technologies) without any supplements. All incubation steps were performed at RT and protected from light. The transfection complex mixtures were added drop-wise to the cells and the plates were gently swirled to disperse the suspension. To non-transfected control cells, the respective amount of serum-free medium without any supplements was added. Cells were incubated at 37 °C, 5% CO_2_ until analysis.

### Electroporation of CD133^+^ HSPCs and K562 cells

Cells were electroporated using the CD34 Cell Nucleofector Kit and Nucleofector II device from Amaxa (Lonza) according to the manufacturer’s protocol with slight modifications. In brief, 0.5–2 × 10^5^ CD133^+^ cells or 5 × 10^5^ K562 cells per sample were pelleted for 5 min at 300 × g and resuspended in 10 μL of Nucleofector Solution (pre-warmed to room temperature). SiRNA at a final concentration of 0.6–2 μM or 8 μg of GFP-mRNA was added in a maximum volume of 10 μL per sample. For mock-electroporation, the equal volume of water instead of mRNA solution was added. Cell suspension was transferred into a cuvette and electroporated with program U-008 for HSPCs or T-016 for K562 cells. After electroporation, 500 μL of the respective pre-warmed culture medium was added to the cuvette. HSPC suspension was transferred into a 24-Well plate containing 100 μL of pre-warmed medium using plastic pipettes provided with the kit in order to avoid sheering forces. K562 cells were transferred into a 12-Well plate with 1.5 mL of medium. Medium was changed after 24 h.

### qRT-PCR

A minimum of 40,000 viable cells per sample was harvested 48 h after transfection. For cDNA synthesis, the μMACS One-step cDNA Kit (Miltenyi Biotec) was used according to the manufacturer’s instructions. qPCR was performed using the qPCR Core Kit for SYBR Green I (Eurogentec). Volume of the reaction mix was scaled down to 20 μL. Primer sequences (Metabion): CD45 fwd:TGGAGGACACAGCACATTGG, rev:TGGAGGACACAGCACATTGG, CD133 fwd:TAGACATTATCTGCAGTGGATCG, rev:GCTACACAGAAAGACATCAACAGCA. GADPH and HPRT were used as housekeeping gene for calculation of CD45 and CD133 knockdown, respectively.

### Cycloheximide assay

CD133^+^ HSPCs or K562 cells were cultured for up to 48 h in the respective culture medium containing 100 μg/mL of cycloheximide (Sigma-Aldrich, ready-made solution in DMSO), and protein expression was determined by flow cytometry. Control cells were cultivated after addition of the corresponding volume of DMSO instead of CHX solution.

### Immunocytochemistry

For siRNA localisation studies, cell surface was stained with CD34-PE antibody (Miltenyi Biotec, clone: AC136). After washing with PBS, cells were fixed and permeabilised in suspension using the Inside stain Kit (Miltenyi Biotec) mainly following the manufacturer’s instruction. Inside fix was used without dilution and cells were pelleted by centrifugation for 5 min at 600 × g. All labelling steps were performed at RT. Rabbit-anti-Rab5b antibody staining (Santa Cruz, sc-598) was performed using a 1:50 dilution for 30 min following incubation with anti-rabbit-IgG-AF594 antibody (Life technologies, A-11012) in a 1:100 dilution for 30 min. For nuclear counterstaining, DRAQ5 (ebioscience) was added at a final concentration of 1.5 μM and incubated for 10 min. Cells were seeded into 8 well CytoCapture chambers (Miltenyi Biotec, H20-10) and analysed with a LSM710 confocal microscope (Zeiss) using a 63× immersion objective. Pictures were processed with the Carl Zeiss ZEN 2011 software.

### Synthesis of GFP-mRNA

Template for *in vitro* transcription comprising a T7 promotor followed by a modified 5′-UTR, the coding sequence of enhanced GPF (eGFP from *Mycobacterium tuberculosis H37Rv*; Gene ID: 20473140), the 3′-UTR of the mouse haemoglobin alpha chain and a restriction site was synthesised by DNA 2.0. Plasmid was linearised by endonuclease restriction and used as template for *in vitro* transcription using T7 RNA polymerase. The *in vitro* transcripts were purified using RNeasy Kit (Qiagen), enzymatically capped (*Vaccinia* capping enzyme), and polyadenylated (poly-A polymerase). After DNase I treatment, mRNAs were again purified using RNeasy Kit (Qiagen). RNA concentrations were determined by measuring the absorbance at 260 nm using a Nanodrop spectrophotometer. The length of the *in vitro* transcripts and the polyadenylated mRNAs were monitored by Bioanalyzer electrophoreses on a RNA Nano chip (Agilent).

### Statistical analysis

Statistical analysis was performed with GraphPad PRISM software 6 (GraphPad, La Jolla, CA) using one-way ANOVA or paired two-tailed t-test.

## Additional Information

**How to cite this article**: Diener, Y. *et al*. RNA-based, transient modulation of gene expression in human haematopoietic stem and progenitor cells. *Sci. Rep*. **5**, 17184; doi: 10.1038/srep17184 (2015).

## Supplementary Material

Supplementary Information
