# Supplementary material for: RNA-based, transient modulation of gene expression in human haematopoietic stem and progenitor cells
Source: Sci Rep. 2015 Nov 24;5:17184. doi: 10.1038/srep17184 (PMC4657003; doi:10.1038/srep17184)
Supplement: Supplementary Information [file srep17184-s1.pdf]

Supplementary Information

RNA-based, transient modulation of gene expression in human haematopoietic stem and progenitor cells

Yvonne Diener[1], Marion Jurk[1], Britta Kandil[1], Yeong-Hoon Choi[2], Stefan Wild[1], Ute Bissels[1], Andreas Bosio[1]

1) Miltenyi Biotec GmbH, Bergisch Gladbach, Germany
2) Heart Center of the University of Cologne, Department of Cardiothoracic Surgery, Center of Molecular Medicine Cologne, University of Cologne, Cologne, Germany

Index

Supplementary Figures 2

Figure S1: Expression of CD133 and CD45 after transfection of HSPCs and control cell lines 2

Figure S2: No knockdown at mRNA or protein level after chemical transfection of HSPCs. 3

Figure S3: PLGA nanoparticles as delivery vehicles. 4

Figure S4: GFP-mRNA does not impair viability or affect CD133 expression. 4

Supplementary Table S1: Optimisation of chemical transfection 5

Supplementary Methods........ 6

PLGA nanoparticle production and application........ 6

Supplementary Figures

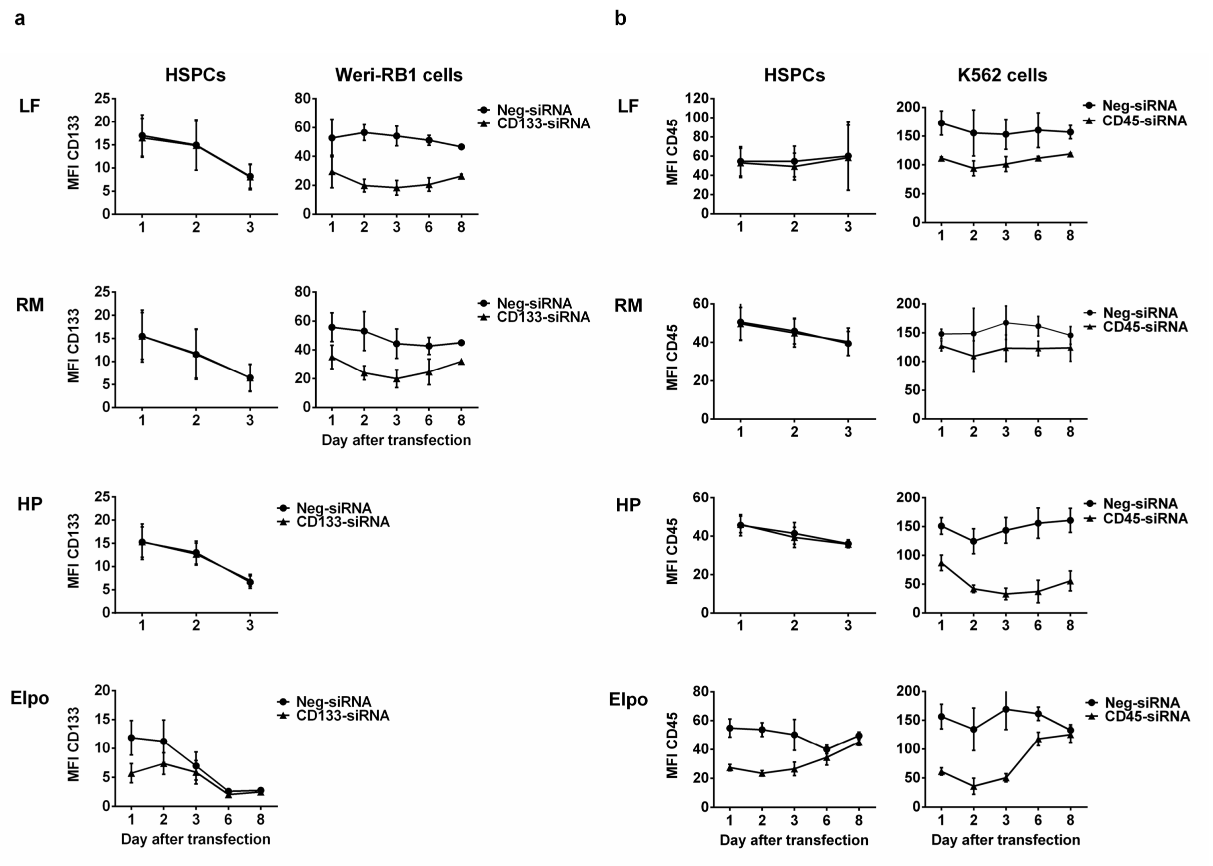

Figure S1: Expression of CD133 and CD45 after transfection of HSPCs and control cell lines. CD133$^+$ HSPCs or indicated cell lines (Weri-RB1, K562) were transfected with siRNAs targeting CD133 (a), CD45 (b) or a non-targeting control siRNA, which has no known target mRNA. Displayed is the median fluorescence intensity (MFI) of the respective surface marker at the indicated time point. LF: Lipofectamine 2000, RM: Lipofectamine RNAiMAX, HP: HiPerFect, Elpo: Electroporation. Mean of three independent experiments is shown for day 1 – 3. For day 6 and 8, mean of two independent experiments is given. Error bars represent SD.

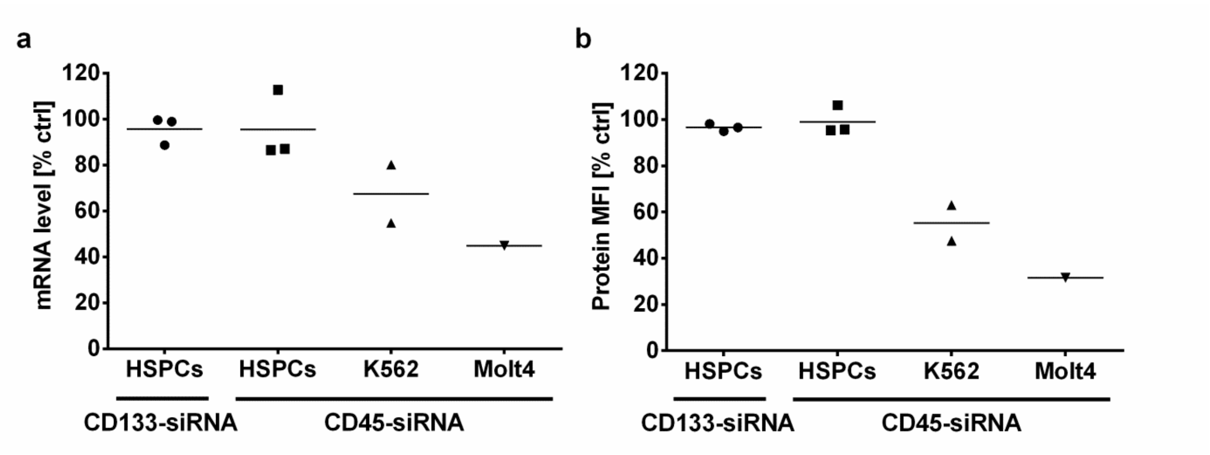

Figure S2: No knockdown at mRNA or protein level after chemical transfection of HSPCs. $CD133^+$ HSPCs or indicated cell lines (K562, Molt4) were transfected with siRNAs targeting CD133 (CD133-siR) or CD45 (CD45-siR) or a non-targeting control siRNA (ctrl). (a) mRNA levels were determined by qRT-PCR 48 h after transfection. (b) Corresponding protein expression was assessed by flow cytometry 48 – 72 h after transfection. Displayed is the median fluorescence intensity (MFI) of the respective surface marker as percentage relative to cells transfected with the control siRNA. Each dot represents one independent experiment.

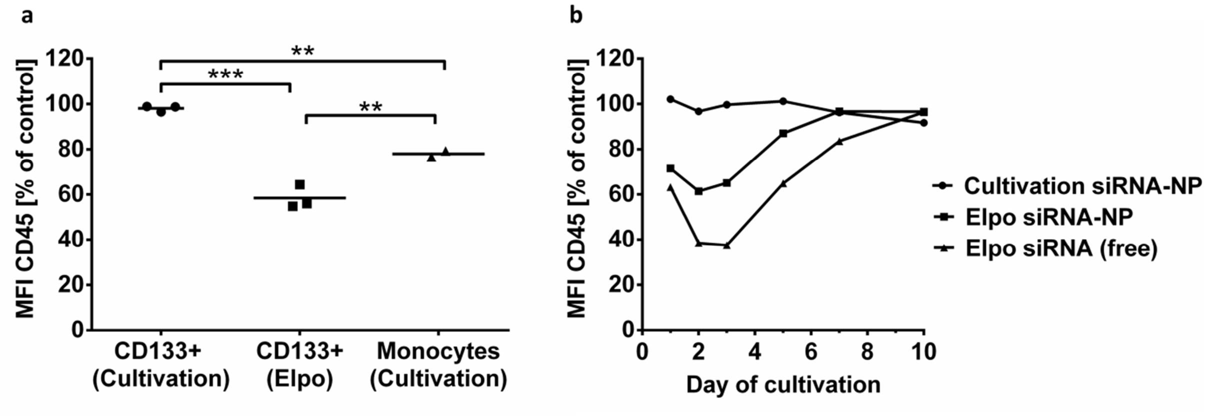

Figure S3: PLGA nanoparticles as delivery vehicles. (a) CD133$^+$ HSPCs or monocytes were cultivated (Cultivation) or electroporated (Elpo) with CD45-siRNA nanoparticles. CD45 expression is displayed as median fluorescence intensity (MFI) relative to control cells (cultivated or electroporated with empty nanoparticles). Dots represent two technical replicates from one experiment and one additional independent experiment for CD133$^+$ cells. (b) Comparison of CD45 knockdown after cultivation or electroporation of CD133$^+$ cells with CD45-siRNA nanoparticles (siRNA-NP) or CD45-siRNA alone (siRNA (free)). Displayed is the MFI of CD45 relative to control cells (cultivated or electroporated with empty nanoparticles or electroporated with non-targeting siRNA) from one experiment. ** P ≤ 0.01, *** P ≤ 0.001, one-way ANOVA.

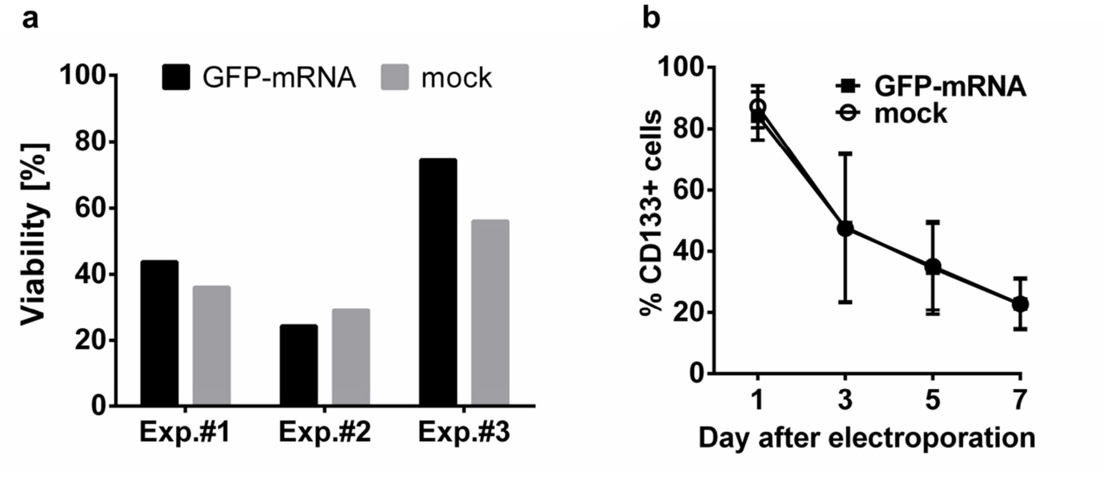

Figure S4: GFP-mRNA does not impair viability or affect CD133 expression. (a) Viability 24 h after electroporation with GFP-mRNA or water (mock) in three independent experiments (Exp. #1–3). Displayed is the number of viable (propidium iodide-negative) cells relative to non-electroporated cells. Each bar represents one independent experiment. (b) Frequency of CD133$^+$ cells among GFP-mRNA– or mock-electroporated cells. Mean of four independent experiments. Error bars represent SD.

Supplementary Table S1: Optimisation of chemical transfection

	Conditions tested				Results		
**Reagent**	**Vol. of Reagent [µl]**	**Final Vol. [µl]**	**Final siRNA conc. [nM]**	**Time [hours after transfection]**	**Efficiency [%AF488+ cells, mean ±SD]**	**Viability [%of untreated cells, mean ±SD]**	**# of replicates**
**HiPerFect**	1.5	175	100	24	97.6 ±1.3	99 ±0.0	5
	1.5	175	50	24	86.3 ±16.1	99 ±1.4	3
	3	175	50	20	70	97	1
	0.75	175	50	20	34	97	1
	0.75	175	100	20	60	97	1
**Lipofecta-mine 2000**	1	150	100	24	76.1 ±12.4	89.2 ±8.5	9
	0.5	150	100	25	48 ±36.8	96.1 ±5.5	2
**RNAiMAX**	0.5	120	100	23	50.1 ±14.6	98.6 ±0.8	7
	0.5	120	50	24	59	98	1
	0.25	120	25	24	18	98	1
	0.25	120	50	24	24	98	1
	0.25	120	100	24	33	98	1
**DOTAP**	2	200	100	24	68.8 ±15.5	70.7 ±22.0	4
	1.4	200	15	24	14 ±9.9	99 ±0.0	2
	1.4	200	50	24	46 ±18.4	95 ±0.0	2
**PEI 2K**	0.8	200	100	26	99 ±0.0	74 ±0.0	3
	0.8	200	100	6	82 ±17.0	87 ±17.0	2
	6.4	200	100	6	98	70	1
**siQuest**	0.3	61	25	19	4.5 ±0.8	99 ±0.0	2
**Dharma-FECT4**	0.25	70	50	19	7	99	1
	0.1	70	50	19	1	99	1
**TKO**	0.5	61	25	19	1	99	1
	0.5	61	50	19	0.5	99	1
**ExGen 500**	1.65	200	100	6	89.3 ±6.0	99 ±0.0	3
	0.8	200	100	6	54	99	1
**ProFection**	1	160	100	18	0	99	1
	0.67	160	50	18	0	99	1

Supplementary Methods

PLGA nanoparticle production and application

Nanoparticles were produced by water-in-oil-in-water (W/O/W) emulsion technique according to McNeer et al[1]. In brief, 100 nmoles of CD45-siRNA (Miltenyi Biotec, target sequence 5´-GGCUUAAACUCUUGGCAUUTT-3´) were complexed with spermidine at a molar ratio of polyamine nitrogen to nucleic acid phosphate of 8:1 (N:P ratio) in 300 µL TE-buffer (10 mM Tris-HCl pH7.4, 1 mM EDTA) for 15 min at RT, according to the protocol of Woodrow and colleagues[2]. The resulting aqueous solution was emulsified into 3 mL of a PLGA solution (Sigma Aldrich, #719897, acid terminated, Mw 7,000–17,000, 50 mg/mL in dichloromethane) for 40 sec using a probe sonicator (Bandelin Sonopuls HD2070, MS72). During this process, a water-in-oil emulsion was formed which was further emulsified into 10 mL of an aqueous solution of 2 % PVA (Sigma Aldrich, #360627, Mw 9,000–10,000) containing 5 % of NaCl (Merck) by an additional sonication for 3 min. The resulting double emulsion was stirred for 18 h at room temperature for the purpose of solvent evaporation. Nanoparticles were collected by centrifugation at 16,000 xg for 40 min, washed twice with deionised water to remove excessive PVA and agent. and then resuspended in deionised water and lyophilised for 25 h. For application to cells, lyophilised particles were resuspended in PBS at 10 mg/mL and sonicated in a water bath for 2 x 10 min to dissolve potential aggregates. The average diameter of the particles was 181 nm with a Polydispersity Index (PDI) of 0.065–0.077, measured in triplicates with the Delsa Nano (Beckman Coulter). For cultivation or electroporation of cells the particles were added at a final concentration of 1 mg/mL to the culture medium or the Nucleofector solution, respectively.

References

1 McNeer, N. A. *et al.* Nanoparticles deliver triplex-forming PNAs for site-specific genomic recombination in CD34+ human hematopoietic progenitors. *Molecular therapy : the journal of the American Society of Gene Therapy* **19**, 172-180, doi:10.1038/mt.2010.200 (2011).

2 Woodrow, K. A. *et al.* Intravaginal gene silencing using biodegradable polymer nanoparticles densely loaded with small-interfering RNA. *Nature materials* **8**, 526-533, doi:10.1038/nmat2444 (2009).
